# Soluble Dietary Fiber from *Polygonatum cyrtonema* Hua Attenuates Cyclophosphamide-Induced Intestinal Injury in Mice

**DOI:** 10.3390/ijms27104537

**Published:** 2026-05-18

**Authors:** Lingqiao Zeng, Shengxin Cui, Teng Peng

**Affiliations:** School of Pharmacy, Chengdu University of Traditional Chinese Medicine, Chengdu 611137, China; zenglingqiao@stu.cdutcm.edu.cn (L.Z.); cuishengxin@stu.cdutcm.edu.cn (S.C.)

**Keywords:** soluble dietary fiber, *Polygonatum cyrtonema* Hua, intestinal injury, intestinal barrier function, mucosal immunity

## Abstract

This study aimed to evaluate the protective effects of soluble dietary fiber (SDF) derived from *Polygonatum cyrtonema* Hua residues on cyclophosphamide (CTX)-induced intestinal injury in mice. A total of 60 C57BL/6 mice (6–8 weeks old; body weight, 23.8 ± 0.5 g) were randomly allocated to six groups (n = 10 per group): a control group (CON), a CTX model group (CTX), a levamisole-treated positive control group (PC), and low-, medium-, and high-dose SDF groups (125, 250, and 500 mg/kg body weight, respectively). Mice received oral administration of SDF or an equal volume of water for 21 consecutive days and were intraperitoneally injected with CTX (80 mg/kg body weight) on days 19–21 to induce intestinal injury. The results demonstrate that SDF possessed a porous, sponge-like network structure and comprised multiple monosaccharides. SDF intervention, particularly at medium and high doses, significantly attenuated CTX-induced body weight loss and immune organ atrophy; restored villus height and the villus-to-crypt ratio; increased the numbers of goblet cells and intraepithelial lymphocytes; elevated intestinal levels of sIgA, β-defensins, and lysozyme; and reduced serum levels of LPS, D-lactic acid, and DAO (*p* < 0.05). In conclusion, SDF derived from *Polygonatum* cyrtonema effectively mitigates CTX-induced intestinal injury by enhancing intestinal mucosal immunity and preserving intestinal barrier integrity, thereby highlighting its potential as a functional ingredient for promoting gut health.

## 1. Introduction

In recent years, changes in dietary patterns and the acceleration of modern lifestyles have led to growing concern regarding intestinal health [1,2]. The intestine serves not only as a critical site for nutrient absorption but also as a key barrier in host immune defense [3,4]. Disruption of intestinal barrier function, oxidative stress imbalance, and gut microbiota dysbiosis are closely associated with the onset and progression of various intestinal diseases and systemic inflammatory responses [5,6,7]. Under conditions of intestinal injury, the structural integrity of the intestinal mucosa is compromised, resulting in increased permeability. This facilitates the translocation of bacteria and their metabolites into the systemic circulation through the compromised barrier, thereby exacerbating inflammatory responses and immune dysfunction [8,9]. Therefore, the development of safe and effective nutritional interventions to maintain intestinal homeostasis has become a major focus of current gut health research.

Currently, aminosalicylates, immunosuppressants, and other anti-inflammatory agents are widely used in clinical practice to alleviate symptoms associated with intestinal inflammation; however, their long-term use is often accompanied by adverse effects, and a proportion of patients remain at risk of relapse [10]. Therefore, the development of natural, safe, and long-term applicable functional dietary components to ameliorate intestinal injury and maintain intestinal barrier function has attracted considerable research interest and exhibits promising application potential [11,12]. As an important component of dietary fiber, soluble dietary fiber (SDF) exhibits excellent water absorption capacity, swelling capacity, water-holding capacity, and fermentability. SDF exerts beneficial effects on intestinal health by modulating the gut microenvironment, promoting the growth of beneficial microbiota, enhancing mucus secretion, and improving intestinal barrier integrity [13,14,15].

*Polygonatum* spp. is a traditional medicinal and edible plant with multiple bioactivities, including immunomodulatory, antioxidant, and hypoglycemic effects, and has been widely applied in functional foods and natural product research [16,17,18]. However, existing studies have primarily focused on its bioactive constituents, such as polysaccharides, whereas the utilization of its by-products remains limited. Notably, extraction residues of *Polygonatum* still contain considerable amounts of SDF, representing a resource with substantial development potential [19]. Although previous studies have demonstrated that dietary fiber can ameliorate intestinal injury by regulating intestinal immunity and barrier function [20,21], systematic investigations into the structural characteristics, compositional features, and protective effects of SDF derived from *Polygonatum* residues against chemically induced intestinal injury remain lacking. Cyclophosphamide (CTX), a widely used chemotherapeutic and immunosuppressive agent, exerts pronounced toxicity on intestinal epithelial and immune cells. It can induce intestinal mucosal injury, increased permeability, and oxidative stress, thereby simulating intestinal barrier dysfunction and inflammatory damage. Therefore, CTX is commonly used to establish experimental models of intestinal injury [22,23,24]. Therefore, in the present study, *Polygonatum* processing residues were used as raw materials for the extraction and purification of SDF. Its morphological and compositional characteristics were subsequently analyzed, and a CTX-induced intestinal injury mouse model was established to systematically evaluate the effects of Polygonatum-derived SDF on intestinal health. This study aims to elucidate the protective role of SDF against intestinal injury and to provide a theoretical basis for the high-value utilization of *Polygonatum* by-products and the development of natural functional materials for gut health interventions.

## 2. Result

### 2.1. Morphology and Composition of SDF from Polygonatum Residues

**Figure 1:**
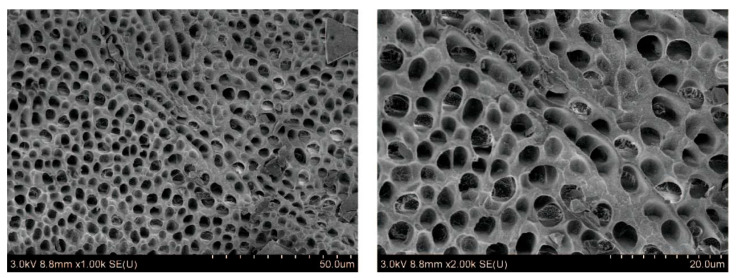
Scanning electron microscopy observation of SDF.

As shown in Figure 1, the SDF displayed a characteristic loose and porous structure, with numerous pores of irregular size and uneven distribution across the surface. The pore walls were relatively thin and interconnected, forming a sponge-like network structure. At higher magnifications, the surface appeared rough and exhibited some degree of aggregation. These morphological features suggest that Polygonatum-derived SDF possesses high porosity, which is conducive to water adsorption and functional performance. Further compositional analysis revealed that SDF is primarily composed of rhamnose, fucose, arabinose, fructose, mannose, glucose, and galactose (Table 1).

### 2.2. Mouse Body Weight

As shown in Table 2, initial body weights did not differ significantly among the groups (*p* = 0.89). At the end of the experiment, changes in body weight differed significantly among the groups (*p* = 0.04). Mice in the CON group attained the highest final body weight, whereas those in the CTX model group exhibited a marked decrease, indicating that CTX treatment substantially inhibited body weight gain. Mice in the PC group and all SDF-treated groups exhibited significantly higher final body weights than those in the CTX group, yet their weights remained lower than the CON group, suggesting that SDF partially mitigated the CTX-induced suppression of body weight gain.

### 2.3. Immune Organ Index in Mice

As shown in Table 3, CTX treatment significantly altered the immune organ indices in mice. Compared with the CON, thymus and spleen indices in the CTX group were significantly reduced (*p* < 0.05), indicating successful induction of immunosuppression by CTX. Compared with the CTX group, all SDF-treated groups exhibited varying degrees of improvement in immune organ indices. The PC group and the medium- and high-dose SDF groups (M, H) exhibited significantly higher thymus indices compared with the CTX group (*p* < 0.05), approaching values observed in the control group. Although the low-dose SDF group exhibited an upward trend, the difference did not reach statistical significance. Similarly, spleen indices in the PC and medium- and high-dose SDF groups were significantly higher than those in the CTX group (*p* < 0.05), with the high-dose group exhibiting the most pronounced recovery.

### 2.4. Histological Morphology of Jejunum and Ileum in Mice

**Figure 2:**
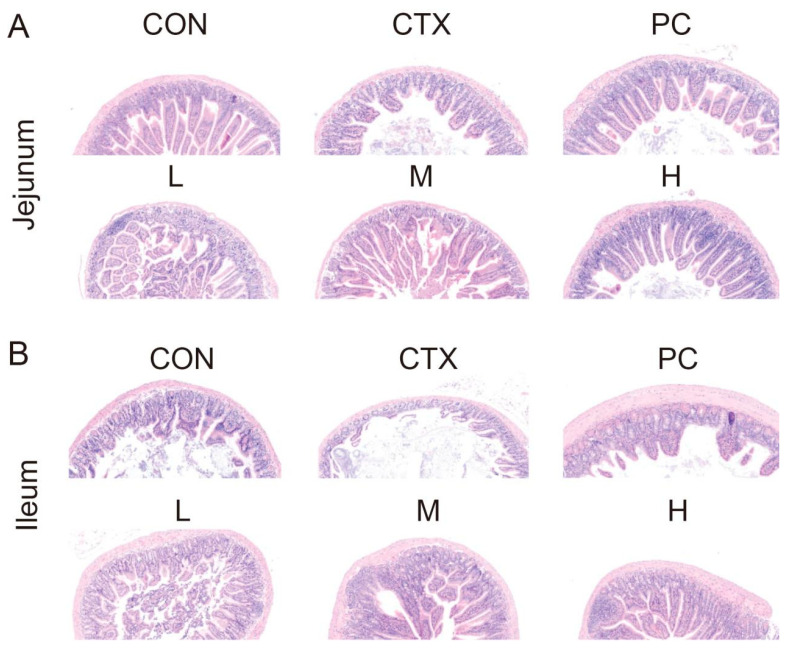
Effect of SDF on the morphology of jejunum (**A**) and ileum (**B**) in mice with intestinal injury. Pink indicates the cytoplasm, and purple indicates the nuclei. (CON) normal control group, (CTX) cyclophosphamide-induced intestinal injury model group, (PC) levamisole hydrochloride-treated CTX group, (L) low-dose SDF group, (M) medium-dose SDF group, (H) high-dose SDF group.

Figure 2A,B depict the histological alterations in the jejunum and ileum of mice across all experimental groups. In the CON group, intestinal architecture was intact, villi were regularly arranged and morphologically uniform, crypt structures were clearly defined, epithelial continuity was preserved, and no evident inflammation or tissue damage was observed. Following CTX treatment, marked intestinal injury was observed. Villi in both the jejunum and ileum displayed varying degrees of atrophy, fragmentation, or shedding. The villi were disorganized, crypt structures were disrupted, and localized epithelial cell loss and tissue loosening were apparent, indicating that CTX effectively induced intestinal barrier damage. Compared with the CTX group, the PC group exhibited marked improvement in intestinal architecture, with partial restoration of villus height and integrity, more orderly villus arrangement, and clearer crypt structures. SDF intervention induced varying degrees of histological restoration in a dose-dependent manner. The low-dose group exhibited limited improvement, with some disorganized villi remaining. The medium-dose group demonstrated notable restoration of villus morphology and relatively regular arrangement. The medium- and high-dose groups exhibited notable improvement, with intestinal architecture approaching that of the control group: villi were intact and orderly, and epithelial continuity was preserved. Collectively, these results indicate that SDF effectively mitigates CTX-induced intestinal injury in mice, with the medium- and high-dose SDF groups conferring more pronounced protection in both the jejunum and ileum.

Furthermore, CTX treatment significantly altered villus and crypt architecture in the small intestine of mice (*p* < 0.05). Compared with the control group, the CTX group exhibited significantly reduced villus height and markedly increased crypt depth, resulting in a decreased villus-to-crypt (V/C) ratio, indicative of impaired intestinal absorptive capacity and disrupted epithelial renewal. In contrast, SDF intervention markedly restored intestinal morphology. Villus heights in the PC and all SDF-treated groups (L, M, H) were significantly greater than those in the CTX group, approaching control levels, while crypt depths were significantly decreased (*p* < 0.05), particularly in the medium- and high-dose groups. The V/C ratio was significantly increased in all intervention groups (*p* < 0.05), with the medium- and high-dose SDF groups exhibiting marked recovery, approaching control values (Table 4). Collectively, these results suggest that SDF alleviates villus atrophy and crypt hyperplasia, thereby contributing to the restoration of intestinal barrier architecture and absorptive capacity.

### 2.5. Goblet Cells and Intraepithelial Lymphocytes in the Small Intestine of Mice

As shown in Table 5, CTX treatment significantly decreased the numbers of goblet cells and intraepithelial lymphocytes (IELs) in the mouse small intestine (*p* < 0.05). In the jejunum, goblet cell numbers in the CTX group were markedly decreased compared with the control group. Similarly, in the ileum, CTX significantly decreased both goblet cell and IEL counts. Following SDF intervention, all treatment groups exhibited varying degrees of restoration. In the jejunum, goblet cell numbers in the PC and all SDF-treated groups (L, M, H) were significantly higher than those in the CTX group (*p* < 0.05). IEL numbers were also significantly increased in the PC group and the medium- and high-dose SDF groups (*p* < 0.05), with the high-dose group exhibiting the most pronounced restoration. In the ileum, SDF treatment similarly increased goblet cell and IEL numbers, particularly in the PC and medium- and high-dose groups, where values were comparable to those of the control group (*p* > 0.05), indicating effective restoration. Collectively, these findings indicate that SDF promotes goblet cell differentiation and facilitates the restoration of intraepithelial lymphocytes in the small intestine.

### 2.6. Intestinal Immune Factors

As shown in Table 6, CTX treatment significantly decreased intestinal immune factor levels in mice (*p* < 0.05). Compared with the CON, levels of sIgA, β-DF, and LZM in the CTX group were markedly decreased, indicative of suppressed intestinal mucosal immunity. Following SDF intervention, levels of intestinal immune factors were elevated across all treatment groups. In the PC and high-dose SDF groups, sIgA levels were restored to values comparable to those of the control group (*p* > 0.05). Similar trends were observed for β-DF and LZM, with the PC and high-dose SDF groups exhibiting significantly higher levels than the CTX group (*p* < 0.05).

### 2.7. Blood Biochemical Indicators

As shown in Table 7, CTX treatment significantly increased blood levels of indicators related to intestinal barrier injury in mice (*p* < 0.05). Compared with the control group, levels of LPS, D-LA, and DAO in the CTX group were markedly elevated, indicating increased intestinal permeability and impaired barrier function. SDF intervention significantly reduced these indicators (*p* < 0.05). In PC, L, M, H groups, LPS, D-LA, and DAO levels were markedly decreased. The medium-dose group showed lower levels of LPS and D-LA, while all SDF doses significantly improved DAO levels compared with the CTX group. These results suggest that SDF can effectively reduce blood LPS, D-LA, and DAO levels, thereby alleviating CTX-induced increases in intestinal permeability.

## 3. Discussion

In this study, the SDF displayed a characteristic loose, porous, sponge-like network with a high specific surface area and abundant porosity. These structural features are generally regarded as essential for the physiological functions of dietary fibers [25]. The porous architecture can markedly enhance water absorption and retention, thereby forming a viscous intestinal matrix, slowing chyme transit, and creating a favorable environment for microbial fermentation [26,27]. Furthermore, the high specific surface area promotes interactions with intestinal bile acids, deleterious substances, and microbial metabolites, thereby contributing to the regulation of the gut microenvironment [28,29]. Additionally, surface roughness and partial aggregation, potentially resulting from intermolecular hydrogen bonding and structural rearrangement during drying, may also affect the solubility and bioavailability of SDF [30]. Previous studies have shown that the composition and relative proportions of individual monosaccharides critically determine the biological activity of polysaccharides [31]. For example, arabinose and galactose are commonly associated with arabinogalactan structures, which promote the proliferation of beneficial bacteria, including *Bifidobacterium* and *Lactobacillus* [32,33]. Rhamnose and fucose are frequently found in polysaccharides with immunomodulatory properties and may contribute to the regulation of intestinal immune responses [34,35]. The presence of mannose may relate to the energy supply and fermentation characteristics of polysaccharides [36]. The complex monosaccharide composition of this SDF likely endows it with multiple biological functions.

In this study, CTX administration markedly suppressed body weight gain in mice and decreased thymus and spleen indices. This observation aligns with the established cytotoxic effects of CTX, which inhibit hematopoiesis and lymphocyte proliferation, indicating that CTX not only impairs systemic immunity but also indirectly restricts growth and development [37,38]. Intervention with SDF significantly mitigated CTX-induced growth retardation and restored thymus and spleen indices, particularly at medium and high doses, nearing those of the control group. These findings suggest that SDF may protect growth performance by enhancing host immune function, promoting nutrient absorption, and supporting overall metabolic homeostasis. CTX administration also caused pronounced disruption of villus and crypt architecture in the jejunum and ileum, indicating severe compromise of intestinal barrier function. A reduced villus-to-crypt ratio further reflected diminished absorptive surface area and disrupted epithelial renewal [39]. SDF supplementation enhanced intestinal protection, likely owing to its soluble fiber properties, which promote mucus secretion, support epithelial nutrient supply, and provide substrates for gut microbiota, thereby preserving intestinal structural integrity [40]. Furthermore, SDF intervention restored the numbers of goblet cells and IELs, particularly in the medium- and high-dose groups, approaching levels observed in control mice. In the present study, restoration of goblet cells and IELs following SDF intervention suggests improved intestinal mucosal barrier function and local immune defense. Goblet cells are specialized epithelial cells responsible for mucus secretion, which forms a protective physical barrier against luminal pathogens and harmful substances, whereas IELs are important immune cells involved in epithelial immune surveillance and defense [41]. Therefore, recovery of goblet cell and IEL numbers may contribute to the maintenance of epithelial integrity and intestinal immune homeostasis. These findings are consistent with previous reports demonstrating that dietary fibers enhance intestinal epithelial immune function [42,43,44,45].

CTX administration markedly reduced intestinal immune factor levels in mice, consistent with its established immunosuppressive mechanism. CTX can compromise the function of intestinal epithelial and mucosal immune cells, leading to decreased mucus secretion and reduced antimicrobial peptide expression, thereby weakening the local intestinal immune barrier and permitting bacterial metabolites or endotoxins to enter circulation, further compromising barrier integrity [22,46,47]. DAO is an intracellular enzyme primarily localized within mature villous epithelial cells of the small intestine. Under normal conditions, the release of DAO from intact epithelial cells is limited, and serum DAO activity remains low. However, when the integrity of the intestinal mucosa is compromised, DAO is released into the circulation. Therefore, serum DAO is widely regarded as a sensitive marker reflecting epithelial cell damage and increased intestinal permeability [48,49]. Meanwhile, D-LA, a bacterial metabolite, is minimally absorbed under normal intestinal barrier conditions. Disruption of tight junction integrity facilitates its translocation into the circulation, rendering it an additional reliable marker of intestinal permeability. In this study, reductions in DAO and D-LA levels following SDF intervention directly reflected the restoration of intestinal epithelial integrity and barrier function in CTX-treated mice [49,50]. Restoration of sIgA suggests that SDF promotes plasma cell differentiation and mucosal immune responses, thereby enhancing luminal defense against pathogens [51,52]. Elevated levels of β-DF and LZM indicate activation of innate immune mechanisms and enhanced antimicrobial defense [53]. Improvements in serum biochemical parameters further demonstrate that SDF preserves epithelial structure, reduces intestinal permeability, and prevents endotoxin translocation into systemic circulation, thereby alleviating systemic inflammation [54]. Notably, the increase in sIgA, β-DF, and LZM observed in this study represents direct experimental evidence of improved mucosal immune function, whereas the mechanistic interpretation that these factors enhance host defense capacity is supported by previous reports. Collectively, SDF enhances mucosal immunity, fortifies local defenses, preserves intestinal barrier integrity, and limits endotoxin translocation, synergistically maintaining intestinal homeostasis.

Although this study systematically evaluated the protective effects of SDF against CTX-induced intestinal injury in mice, the intervention doses and durations were relatively fixed, and the potential influence of varying doses or treatment durations on intestinal protection was not investigated, potentially overlooking dose- or time-dependent effects. Furthermore, key parameters, including gut microbiota composition and microbial metabolites, were not comprehensively evaluated, limiting a more in-depth understanding of the mechanisms underlying SDF action. These limitations suggest that future studies should incorporate multiple experimental models, dose–response designs, and multi-level parameter analyses to further elucidate the mechanisms by which SDF protects the intestine and to explore its potential applications.

## 4. Materials and Methods

### 4.1. Preparation of Polygonatum cyrtonema Hua Residues

Fresh rhizomes of *Polygonatum cyrtonema* Hua were obtained from a local herbal medicine market in Chengdu, Sichuan Province, China. The plant materials were subsequently used for water extraction to obtain extraction residues. The resulting extraction residues were oven-dried at 60 °C until completely dry. The dried extraction residues were then ground and passed through an 80-mesh sieve for subsequent use.

### 4.2. Extraction of Soluble Dietary Fiber from Polygonatum Residues

Soluble dietary fiber was extracted from the *Polygonatum* extraction residues under the following conditions: a material-to-liquid ratio of 1:30, α-amylase at 1.5%, papain at 0.15%, and cellulase at 0.20%. Under these conditions, the yield of SDF reached 19.20% and exhibited good reproducibility.

### 4.3. Purification of Soluble Dietary Fiber from Polygonatum Residues

DEAE Sepharose Fast Flow resin (GE Healthcare, Uppsala, Sweden) was pretreated with 0.5 M HCl for 1 h and thoroughly washed to remove impurities. The column resin was subsequently rinsed with 4–5 column volumes of distilled water until neutrality was achieved. The peristaltic pump flow rate was set to 5 mL/min, and the column was equilibrated with distilled water for 2 h. Precisely weighed 1 g of crude SDF was dissolved in water and centrifuged to obtain the supernatant, which was then applied to a DEAE Sepharose Fast Flow ion-exchange column (700 mm × 16 mm). The peristaltic pump flow rate was subsequently increased to 15 mL/min, and the sample was sequentially eluted with three column volumes of distilled water, namely 0.2 M NaCl, 0.5 M NaCl, and 1.0 M NaCl. The carbohydrate content of each fraction was determined using the phenol–sulfuric acid method at 490 nm, and fractions were collected accordingly based on the measurement. Collected fractions were concentrated and dialyzed (MWCO 3500 Da, Sigma-Aldrich, St. Louis, MI, USA), followed by lyophilization. Fractions exhibiting higher yield and total sugar content were further purified using gel column chromatography. For this purpose, 100 mg of the fraction was dissolved in 3 mL of distilled water and centrifuged at 12,000 rpm for 10 min, and the resulting supernatant was applied to the gel column. Eluates were automatically collected, followed by dialysis, concentration, and freeze-drying to obtain the final SDF purified via gel column chromatography.

### 4.4. Scanning Electron Microscopy Observation of SDF from Polygonatum Residues

The samples were mounted onto double-sided adhesive tape and observed using a scanning electron microscope (Hitachi SU8010, Tokyo, Japann) at an accelerating voltage of 3.00 kV. Images were captured at magnifications corresponding to 50 μm and 20 μm scales.

### 4.5. Component Analysis of SDF from Polygonatum Residues

The composition of SDF was analyzed by high-performance liquid chromatography coupled with an evaporative light scattering detector (HPLC–ELSD; Agilent 1260 Infinity II, Waldbronn, Germany). Chromatographic separation was performed on an Agilent ZORBAX Original 70 Å Carbohydrate Analysis column (4.6 mm × 250 mm, 5 μm; Agilent, Santa Clara, CA, USA) under the following conditions: column temperature of 30 °C, mobile phase acetonitrile–water (85:15, *v*/*v*), and flow rate of 1.0 mL·min^−1^. Parameters were set as follows: drift tube temperature of 75 °C, gas flow rate of 2.5 L·min^−1^, and gain of 1. Standard solutions were precisely injected at volumes of 2 μL and 10 μL, whereas sample solutions were injected at 10 μL. Quantification was performed using an external two-point calibration method, with concentrations calculated based on a logarithmic equation.

### 4.6. Animal Experiments and Sampling

#### 4.6.1. Experimental Design

Following a 7-day acclimation period, a total of 60 C57BL/6 mice (male, 6–8 weeks old; body weight, 23.8 ± 0.5 g) were randomly assigned to six groups (n = 10 per group): normal control group (CON), cyclophosphamide (CTX)-induced intestinal injury model group (CTX), levamisole hydrochloride-treated CTX group (PC), low-dose SDF group (L), medium-dose SDF group (M), and high-dose SDF group (H). From day 1 to day 21 following the acclimation period, the three SDF groups were orally administered 125, 250, and 500 mg/kg body weight (bw) of SDF each morning, respectively, whereas the control and model groups received an equal volume of distilled water. Starting on day 19, mice in the CTX, PC, and SDF-treated groups were intraperitoneally injected with CTX (purity > 98%, Sigma-Aldrich, St. Louis, USA) at 80 mg/kg body weight once daily for three consecutive days. Mice in the normal control group received an equal volume of saline solution. Throughout the experiment, food and water were provided ad libitum. The experimental design is summarized in Table 8.

#### 4.6.2. Sample Collection and Analysis

During the experimental period, mice were weighed daily using an electronic balance before oral administration, for a total of 21 days. On day 22, mice were fasted for 12 h (water available) prior to weighing. Twenty-four hours after the last dose, all animals were fasted for 12 h, and blood was collected via orbital puncture. Mice were then euthanized by cervical dislocation. Fresh blood samples were collected into additive-free tubes, allowed to stand at room temperature (26 °C) for 30 min, and centrifuged at 3000× *g* for 10 min at 4 °C. The resulting serum was separated and stored at −80 °C. Sections of the jejunum and ileum (3–5 cm each) were excised and fixed in general-purpose tissue fixative for further analysis.

#### 4.6.3. Immune Organ Index

The thymus and spleen of each mouse were excised and weighed. The spleen index and thymus index were calculated using the following formulas:

Spleen index (mg/g) = Spleen weight (mg)/Body weight (g)

Thymus index (mg/g) = Thymus weight (mg)/Body weight (g)

#### 4.6.4. Morphological Observation of Jejunum and Ileum Tissues

Following dissection, jejunum and ileum samples were fixed in 4% paraformaldehyde for 48 h, followed by graded ethanol dehydration and paraffin embedding. The paraffin-embedded blocks were sectioned, deparaffinized, and mounted onto slides for hematoxylin–eosin (HE) staining. Sections were stained with hematoxylin for 4 min, differentiated in acidified water and ammonia water for 30 s, rinsed under running water for 60 min, and sequentially dehydrated in 80% and 95% ethanol for 5 min each. Sections were then stained with eosin for 4 min. The stained sections were subsequently dehydrated three times in absolute ethanol (5 min each) and cleared in xylene three times (5 min each), before being mounted with neutral resin. Jejunum and ileum tissue morphology was observed under a light microscope (Olympus CX43, Tokyo, Japan). For quantitative analysis, five of the longest and intact villi in each microscopic field were selected to measure villus height and crypt depth.

#### 4.6.5. Quantification of Goblet Cells and Intraepithelial Lymphocytes in the Small Intestinal Epithelium

Following dissection, jejunum and ileum tissues were fixed in 4% paraformaldehyde for 24 h, dehydrated, and processed using standard paraffin embedding procedures, followed by hematoxylin–eosin (HE) staining. Under a light microscope, five of the longest and intact villi in each microscopic field were selected for analysis. The number of goblet cells within the selected villi was counted, and intraepithelial lymphocytes (IELs) were quantified per 100 columnar epithelial cells of the villi.

#### 4.6.6. Serum and Tissue Biochemical Analysis

Whole blood collected from mice was centrifuged at 3000× *g* for 15 min at 4 °C to obtain serum samples. Jejunum tissues were thawed on ice, flash-frozen in liquid nitrogen, ground into a fine powder, and collected in Eppendorf tubes. A weighed amount of jejunal tissue powder was suspended in 15 volumes of pre-chilled RIPA lysis buffer (strong) and incubated on ice for 30 min. The lysates were subsequently centrifuged at 14,000 rpm for 15 min at 4 °C, and the supernatants were collected as jejunal tissue homogenates. Protein concentrations in the tissue homogenates were determined using a BCA protein assay kit (Beyotime, Shanghai, China) according to the manufacturer’s instructions. Levels of serum diamine oxidase (DAO), D-lactic acid (D-LA), and lipopolysaccharide (LPS), as well as jejunal tissue homogenate secretory IgA (sIgA), β-defensins (β-DF), and lysozyme (LZM), were quantified using ELISA kits (Nanjing Jiancheng Bioengineering Institute, Nanjing, China) in strict accordance with the manufacturer’s protocols.

#### 4.6.7. Statistical Analysis

All statistical analyses were performed using SPSS Version 21.0 (IBM Corp., Armonk, NY, USA). Normality and homogeneity of variance were assessed before group comparisons. For normally distributed data with homogeneous variance, one-way analysis of variance (ANOVA) followed by Tukey’s post hoc test was used to compare differences among groups. For non-normally distributed data, the Kruskal–Wallis test followed by Dunn’s multiple-comparison post hoc test was applied. A *p*-value ≤ 0.05 was considered statistically significant.

## 5. Conclusions

In summary, SDF derived from Polygonatum residues exerts potential protective effects on the intestine. Structurally, SDF exhibits a loose, porous, sponge-like architecture and is predominantly composed of diverse monosaccharides, providing a structural basis for its bioactivity. In the CTX-induced intestinal injury model, SDF intervention markedly alleviated body weight loss and reductions in immune organ indices; mitigated villus atrophy, crypt hyperplasia, and tissue disorganization in the jejunum and ileum; promoted recovery of goblet cells and intraepithelial lymphocytes; and enhanced levels of intestinal mucosal immune factors. Additionally, SDF significantly decreased serum levels of LPS, D-LA, and DAO, thereby alleviating heightened intestinal permeability and barrier dysfunction. Collectively, these findings demonstrate that SDF derived from Polygonatum residues may contribute to the alleviation of CTX-induced intestinal injury and the maintenance of intestinal homeostasis, providing a theoretical foundation for the high-value utilization of by-products from *Polygonatum* processing and the development of functional materials for intestinal health interventions.

## Figures and Tables

**Table 1:** Composition of SDF.

Ingredient	Rhamnose	Fucose	Arabinose	Fructose	Mannose	Glucose	Galactos
Concentration/(g/kg)	5.37	22.77	63.85	4.05	46.87	3.69	59.91

**Table 2:** Effect of SDF on body weight in mice with intestinal injury.

Group	CON	CTX	PC	L	M	H	SEM	*p*-Value
Initial body weight/g	24.23	23.89	24.32	23.77	24.5	23.96	0.98	0.89
Final body weight/g	34.56 ^a^	31.23 ^c^	33.32 ^ab^	32.12 ^ab^	32.27 ^ab^	32.89 ^ab^	1.02	0.04

^a–c^ Means within a row followed by different lower-case letters differ significantly from each other (*p* < 0.05).

**Table 3:** Effect of SDF on immune organ indices in mice with intestinal injury.

Group	CON	CTX	PC	L	M	H	SEM	*p*-Value
Thymus index (mg/g)	1.53 ^a^	0.85 ^b^	1.31 ^a^	1.21 ^ab^	1.26 ^a^	1.35 ^a^	0.05	0.01
Spleen index (mg/g)	3.45 ^a^	2.03 ^b^	2.89 ^a^	2.45 ^ab^	2.65 ^a^	2.78 ^a^	0.06	0.03

^a,b^ Means within a row followed by different lower-case letters differ significantly from each other (*p* < 0.05).

**Table 4:** Effect of SDF on villi and crypts in mice with intestinal injury.

Group	CON	CTX	PC	L	M	H	SEM	*p*-Value
Villus heigh/µm	400.23 ^b^	310.12 ^a^	380.56 ^b^	385.56 ^b^	390.34 ^b^	386.54 ^b^	10.23	0.01
Crypt depth/µm	100.55 ^b^	118.98 ^a^	108.56 ^b^	105.65 ^b^	104.34 ^b^	112.21 ^b^	5.45	0.02
Villus/crypt	3.78 ^b^	2.69 ^a^	3.52 ^b^	3.12 ^b^	3.23 ^b^	3.53 ^b^	0.54	0.01

^a,b^ Means within a row followed by different lower-case letters differ significantly from each other (*p* < 0.05).

**Table 5:** Effect of SDF on the number of goblet cells and intraepithelial lymphocytes in the small intestine of mice with intestinal injury.

Intestinal	Group	CON	CTX	PC	L	M	H	SEM	*p*-Value
Jejunum	Goblet cells	12.35 ^a^	6.89 ^c^	10.24 ^ab^	9.89 ^ab^	9.99 ^ab^	10.23 ^ab^	0.89	0.01
	Intraepithelial lymphocytes	12.27 ^a^	7.56 ^c^	10.78 ^ab^	8.54 ^abc^	9.46 ^ab^	10.35 ^ab^	0.89	0.02
Ileum	Goblet cells	7.88 ^a^	5.45 ^b^	6.89 ^a^	6.45 ^ab^	6.78 ^a^	7.01 ^a^	0.45	0.03
	Intraepithelial lymphocytes	5.26 ^a^	3.44 ^b^	4.89 ^a^	4.77 ^ab^	4.98 ^a^	5.01 ^a^	0.28	0.01

^a–c^ Means within a row followed by different lower-case letters differ significantly from each other (*p* < 0.05).

**Table 6:** Effect of SDF on intestinal immune factors in mice with intestinal injury.

Group	CON	CTX	PC	L	M	H	SEM	*p*-Value
sIgA/(g/mL)	8.87 ^a^	6.77 ^b^	8.66 ^a^	7.86 ^ab^	7.65 ^ab^	7.98 ^a^	0.74	0.02
β-DF/(µg/mL)	43.15 ^a^	37.02 ^b^	41.23 ^a^	38.65 ^ab^	38.78 ^ab^	39.02 ^a^	1.65	0.02
LZM/(µg/mL)	16.45 ^a^	10.24 ^b^	14.89 ^a^	12.35 ^ab^	13.45 ^ab^	14.02 ^a^	1.15	0.01

^a,b^ Means within a row followed by different lower-case letters differ significantly from each other (*p* < 0.05).

**Table 7:** Effect of SDF on blood biochemical parameters in mice with intestinal injury.

Group	CON	CTX	PC	L	M	H	SEM	*p*-Value
LPS (ng/mL)	200.12 ^b^	240.56 ^a^	199.15 ^b^	189.12 ^b^	185.65 ^b^	195.68 ^b^	6.45	0.03
D-LA (ng/mL)	38.48 ^b^	42.32 ^a^	37.56 ^b^	38.12 ^b^	36.89 ^b^	37.99 ^b^	1.2	0.02
DAO (pg/mL)	312.12 ^b^	489.56 ^a^	350.23 ^b^	355.65 ^b^	375.65 ^b^	360.25 ^b^	12.54	0.04

^a,b^ Means within a row followed by different lower-case letters differ significantly from each other (*p* < 0.05).

**Table 8:** Animal experimental design.

Group	Days 1–21	Days 19–21
CON (Control)	Distilled water	Saline
CTX	Distilled water	80 mg/kg CTX
PC (Positive Control)	10 mg/kg Levamisole hydrochloride	80 mg/kg CTX
L	125 mg/kg SDF-1	80 mg/kg CTX
M	250 mg/kg SDF-1	80 mg/kg CTX
H	500 mg/kg SDF-1	80 mg/kg CTX

(CON) normal control group, (CTX) cyclophosphamide-induced intestinal injury model group, (PC) levamisole hydrochloride-treated CTX group, (L) low-dose SDF group, (M) medium-dose SDF group, (H) high-dose SDF group.

## Data Availability

The raw data supporting the conclusions of this article will be made available by the authors on request.
